# Parasitological Survey in European Brown Hare (*Lepus europaeus Pallas, 1778*) Breeding Facilities in Southern Italy

**DOI:** 10.3390/pathogens12020208

**Published:** 2023-01-29

**Authors:** Leonardo Brustenga, Maria Pia Franciosini, Manuela Diaferia, Giulia Rigamonti, Laura Musa, Barbara Lidia Russomanno, Fabrizia Veronesi

**Affiliations:** 1 Department of Veterinary Medicine, University of Perugia, Via San Costanzo, 6, 06126 Perugia, Italy; 2 Freelance Veterinarian, Vienna Street 26, 85100 Potenza, Italy

**Keywords:** *Lepus europaeus*, endoparasites, captive bred animals, Southern Italy

## Abstract

Parasites are considered important regulating factors of hosts’ population dynamics, not only in free-ranging wildlife, but also in captive bred animals. To date, only few studies have been carried out to assess the parasitic communities of the European brown hare in Southern Italy, and only one focused on animals in captivity. The aim of the present survey was to assess the composition of the endoparasite community in game hares bred for restocking purposes. For this purpose, 215 fecal pools collected in eight different breeding facilities were examined by qualitative and quantitative coprological techniques. Parasites characterized by a direct life cycle, including six species of coccidia from the genus *Eimeria* and the nematode *Trichostrongylus retorataeformis*, proved to be the most prevalent parasites. Further helminthic infestations by *Passalurus ambiguous*, *Strongylosides papillosus*, *Cittotenia* spp. and *Dicrocoelium dendriticum* were also detected, but with an overall prevalence lower than 20%. The present study contributes to increasing knowledge on the health status of a poorly investigated species, and is useful for optimizing breeding efforts in captivity.

## 1. Introduction

The European brown hare (*Lepus europaeus Pallas*, 1778; hereafter hare) is the leporid with the widest global distribution [1]. Italian populations were significantly altered starting from the twentieth century by its restocking with captive-bred or allochthonous animals in order to maintain viable populations for hunting purposes [2]. According to the latest (2013) species assessment of the Italian nucleus of the International Union for the Conservation of Nature (IUCN), the hare suffered a population decline caused mainly by habitat degradation due to new agricultural practices and overexploitation caused by hunters and predators. Since the beginning of the 1990s, better management practices and frequent restocking have reversed the declining trend of the Italian population (http://www.iucn.it/scheda.php?id=-2089037830, accessed on 2 December 2022). Since the hare represents the most important small game species in Europe [3], several studies on the parasitic biocenoses of wild and captive animals were produced across Europe [4,5,6,7,8,9]. Panayotova–Pencheva [10] published an overview of the endoparasites of hares, comparing evidence gathered from 23 countries. Detecting and controlling parasitic diseases is of utmost importance in captive-breeding projects, as parasitic infestations can have a negative impact on the dynamics of populations [11]. Evidence shows that lungworms and gastro-intestinal parasites can affect health status, impact the fitness of animals [6] and that a condition of polyparasitism can often lead to the exacerbation of the pathological effects of the single parasitic species [5]. Moreover, the translocation of non-native parasites in a population of naïve hosts can dramatically impact their health and, consequently, their ecology [12], potentially undermining conservation efforts. The majority of parasitological assessments on hares in Italy were carried out in Northern and Central Italy and mainly focused on free-ranging animals. Few surveys have been carried out on intensely and semi-intensely reared animals [8,11,13,14], and only one was performed in Southern Italy on captive-bred hares [15]. Therefore, an evaluation of the endoparasites of captive bred hares designed for the conservation and support of local nuclei of *Lepus europaeus* was mandatory.

The aim of the present study was to carry out a parasitological survey on captive breeding populations of brown hares reared in facilities of Southern Italy in order to assess their health status and the need of a preventive medicine approach against parasites.

## 2. Materials and Methods

### 2.1. Animals and Coprological Sampling

The survey was carried out within 4 months (from October to February) in eight *L. europaeus* breeding facilities (coded from A1 to A8); animals were housed in cage-free enclosures in which they were kept from the age of 8 weeks. Enclosures hosted from 7 to 40 animals, depending on enclosure size, food availability and water supply. The only antiparasitic treatment administered consisted of robenidine hydrochloride-based medicated pellet added to the hay, starting in the period in which the animals were housed and lasting until the time of the release.

Sampling was carried out on a total of 644 reared animals. The number of animals and density in each facility consisted of respectively: 80, 40 animals/ha (A1); 64, 12 animals/ha (A2); 96, 19 animals/ha (A3); 116, 12 animals/ha (A4); 91, 14 animals/ha (A5); 63, 21 animals/ha (A6); 120, 10 animals/ha (A7); 15, 15 animals/ha (A8). Fecal pools were collected proportionally to the number of animals housed in each enclosure, keeping a ratio of one sample for every three hares. Two hundred and fifteen pools (A1:27; A2:21; A3:32; A4:39; A5:30; A6:21; A7:40; A8:5) consisting of 15–20 g of fresh feces were collected in different areas of the enclosure and promptly refrigerated for no more than 72 h before to be processed.

### 2.2. Coprological Investigations

The fecal pools were submitted to qualitative copromicroscopic analyses by using two flotation solutions with low (50% ZnCl_2_, s.g. 1300) and high density (K_2_HgI_4_, s.g. 1450), according to the procedure described by Dryden et al. [16]. The intensity of shedding was estimated by using the McMaster technique, setting a cut-off of 50 eggs per gram of feces (EPG)/50 oocysts per gram of feces (OPG) [17]. Fecal samples that tested positive for oocysts were also treated with 2.5% K_2_Cr_2_O_7_ to allow oocysts sporulation in order to taxonomically identify the coccidian species, based on the taxonomic identification keys [18,19]. The Baermann technique was used to detect the first larval stages (L_1_) of lungworms.

### 2.3. Statistical Analysis

The overall and relative prevalence of each parasite species and related 95% confidence intervals (C.I. 95%) were calculated by using Microsoft Excel software, as well as the average intensity of shedding of EPG/OPG with the relative range (minimum–maximum). The association between the infections and the variable “breeding facility” was assessed by using a Chi-square test, setting the statistical significance at *p*-value < 0.05.

## 3. Results

The coprological examinations showed that coccidia of the genus *Eimeria* were the most prevalent parasites, followed by helminthic species, including *Trichostrongylus retortaeformis*, *Strongyloides papillosus*, *Passalurus ambiguus*, *Cittotenia* spp. and *Dicrocoelium dendriticum*. It is noteworthy that at least three different species of coccidia and the nematode *T. retortaeformis* were detected in each of the eight analyzed facilities.

**Figure 1:**
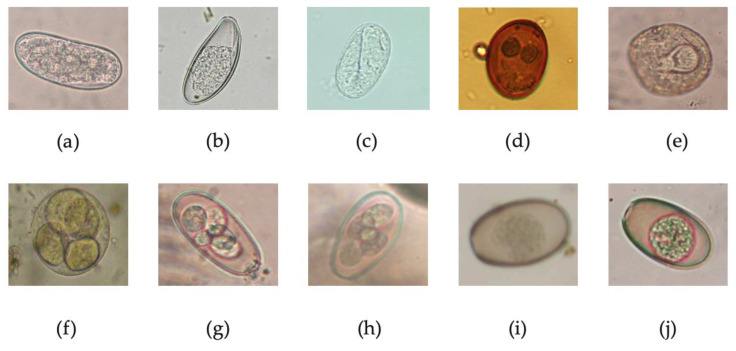
Eggs of helminths (**a**–**e**) and coccidia (**f**–**j**) detected in the survey. (**a**) *Trichostrongylus retortaeiformis* egg; (**b**) *Passalurus ambiguous* egg; (**c**) *Strongyloides papillosus* egg; (**d**) *Dicrocoelium dendriticum* egg; (**e**) *Cittotenia* sp. egg; (**f**) *Eimeria hungarica* sporulated oocyst; (**g**) *Eimeria leporis leporis* sporulated oocyst; (**h**) *Eimeria europaea* sporulated oocyst; (**i**) *Eimeria townsendi* oocyst; (**j**) *Eimeria robertsoni* oocyst.

The results obtained by the qualitative copromicroscopic analyses are summarized in Table 1 and a panel of pictures of the helminthic eggs and coccidian oocysts is provided in Figure 1.

The overall prevalence for coccidia was 91.2%, with a facility level prevalence ranging from 81.3% to 100%, and an average shedding intensity of 3587.5 OPG (range 1650–6450). The prevalence of coccidia infection was not statistically different among the breeding facilities (*p* = 0.056).

Overall, six species of coccidia from the Genus *Eimeria* were found—*Eimeria europaea*, *Eimeria hungarica*, *Eimeria leporis leporis*, *Eimeria robertsoni*, *Eimeria semisculpta* and *Eimeria townsendi* with different distribution and association among the breeding facilities.

*Eimeria l. leporis* was the predominant coccidian species, with an overall prevalence of 69.3%; it was detected in seven out of the eight breeding facilities, and it was the species at highest prevalence, excluding the breeding facility A2. *Eimeria robertsoni* and *E. townsendi* were also found to be at a high prevalence, i.e., 62.3% and 60%, respectively.

**Figure 2:**
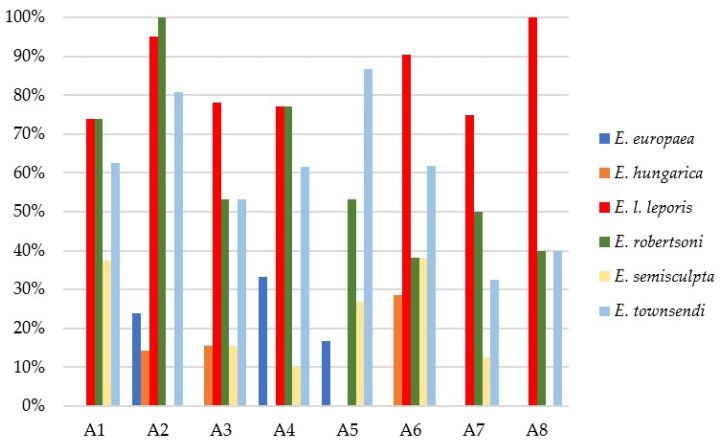
Relative prevalence for each of the six *Eimeria* species detected in the eight hare breeding facilities.

An overview of the relative and overall prevalence of the different coccidian species and the coccidian community within each breeding facility is reported in Table 2 and Figure 2, respectively.

*Eimeria robertsoni* was the predominant species in A2 and showed the same high rates of positivity of *E. l. leporis* in A1 and A4; however, *E. townsendi* was predominant in A5. Other species of coccidia were found with a lower prevalence (<20%), i.e., *Eimeria europaea*, *E. hungarica* and *E. semisculpta*. *Eimeria semisculpta* was detected in six out of the eight breeding facilities; conversely, *E. europaea* and *E. hungarica* were not as widely diffused, being recorded in three out of eight breeding facilities. The coccidian community within the breeding facilities varied and constituted from two to up to five species, with different associations; in particular, the most frequent association detected was between *E. l. leporis* and *E. robertsoni*.

*Trichostrongylus retortaeformis* was the most prevalent helminth detected; it was recorded in all of the analyzed facilities, with an overall prevalence of 21.4%, ranging from 10.3% to 40%, with an average shedding intensity of 497.5 EPG (range 250–900 EPG).

*Strongyloides papillosus* was detected in 50% of the breeding facilities, with an overall prevalence of 6.5% (average shedding intensity 241.5 EPG), ranging from a minimum 5% in A7 to a maximum of 18.5% in A1.

The oxyurid *P. ambiguous* was found in five breeding facilities, with an average prevalence of 9.3% (average shedding intensity 210 EPG), ranging from 5.1% to 23.8%.

Tapeworms from the genus *Cittotenia* were detected in three breeding facilities, with an average prevalence of 5.6% and with an average shedding intensity of 183.5 EPG, whereas the fluke *D. dendriticum* was recorded in only A1 with, a relative prevalence of 11.1% and an average shedding intensity of 200 EPG. Among the helminth infections recovered, only *T. retortaeformis* and *P. ambiguous* showed a statistically different prevalence among the breeding facilities (*p* = 0.02, *p* = 0.019, respectively); no statistically significant differences were recorded for *S. papillosus* (*p* = 0.072), *Cittotenia* spp. (*p* = 0.25) and *D. dendriticum* (*p* = 0.052).

A condition of polyparasitism was recorded in all the breeding facilities. From seven to eight parasite species were simultaneously detected in seven out of the eight breeding facilities. The most common parasitic association consisted of coccidia and *T. retortaeformis* co-infections, present in 100% of the breeding facilities, followed by co-infections with coccidia, *T. retortaeformis* and *P. ambiguous*, detected in 62.5% of the breeding facilities.

The A8 breeding facility showed the lowest species richness, with only three coccidian species (i.e., *E. l. leporis*, *E. robertsoni* and *E. townsendi*) and *T. retortaeformis*.

## 4. Discussion

Investigations such as the one here conducted are extremely important; in fact, although hare breeding has been practiced since ancient times, it is often not very rational and associated with health problems. Attempts to breed hares in enclosures on the ground and in conditions of controlled freedom can fail due to the emergence, in a short time, of various infectious and parasitic diseases, especially those whose pathogens have a long persistence in the soil (i.e., coccidia and strongyles) [20].

Up to date, most of the parasitological investigations reported in Italy were conducted on animals dead of natural causes or killed by hunters, and only few studies were based exclusively on coprological examinations, such as the survey here conducted [11,14]. The analysis of fecal samples collected from the environment might lead to misleading interpretations, due to the risk of sampling the feces of the same animal (for instance, one having a higher coccidia excretory load than others) several times, especially in smaller enclosures. However, this approach is indispensable, since it is not always possible to proceed with the capture or killing of animals kept in captivity for the conservation and support of wild populations.

The results of the present survey are consistent with previously published results obtained in studies carried out at national and international levels [4,5,6,7,8,9,10,11,13,14,15]. The most prevalent parasites were coccidia of the Genus *Eimeria*, with an overall prevalence of 91.2%, representing the highest detected in Italy to the authors’ knowledge, considering both free-ranging and captive-breed populations. An overview of the most relevant national and European data on *Eimeria* spp. infections observed in European brown hare populations is provided in Table 3.

A moderate variation in the prevalence rates of coccidia was observed among the breeding facilities; the highest prevalence rates were not achieved in the facilities housing the greatest number of animals, as expected on the basis of previously available data [22,23]. It could be speculated that such variations might be linked to abiotic environmental factors able to affect the extra-intestinal developmental phase of the parasites (e.g., altitude, exposure to the sun and wind, type of landscape and size of enclosures). In fact, the farms (A2, A4, A5, A8) in which the highest prevalence for coccidia have been recorded (100%) correspond to the more ventilated areas and those at higher altitudes.

The six species of coccidia detected in the present survey were in line with the most frequently found in *L. europaeus* populations across Italy and Central/Western Europe [6,7,8,10,21]. It is important to note the strict species specificity of the identified *Eimeria* species, which, although morphologically very similar to those of other lagomorphs such as *Oryctolagus* and *Sylvilagus*, belongs exclusively to the genus *Lepus* [18,19,24].

Each of the coccidian species were localized in the intestines, and some of them are considered to be at high pathogenicity [25] and might represent important mortality factors [26,27]. In particular, *E. l. leporis* and *E. robertsoni* are described as the cause of severe enteritis of large segments of the small intestine, mostly in juveniles [6], and are demonstrated to be an important cause of mortality in hares [28].

Paoletti et al. [15] reported further intestinal coccidia, i.e., *Eimeria leporis brevis*, *Eimeria deharroi* and *Eimeria tailliezi*, in *L. europaeus* populations from Southern Italy; those species are commonly described in animals from east Europe and South America [15]. The same authors described also the presence of *Eimeria stiedai*, responsible for the hare hepatic coccidiosis and having the highest pathogenicity and rate of mortality.

In spite of the high prevalence of coccidia, no outbreaks of coccidiosis were reported in the anamnestic data, possibly thanks to the use of coccidiostat supplemented in the pellet feed. The coccidiostat supplementation could also explain the quite low shedding intensity observed here. Captive animals generally show higher levels of oocyst excretion than wild subjects, even if constantly lower than those found in reared rabbits [24]. Reared hares commonly have a shedding intensity higher than 10,000 OPG [8,14] with values that may reach 100,000 OPG or even more, especially in animals kept in cages [29]. The quantitative oocyst findings here ranged from 1650 to 6450 OPG, with an average shedding intensity of 3587.5 OPG. However, in order to obtain a correct estimate of the infestation dynamics in the breeding facilities, a longitudinal survey should be conducted, taking into account abiotic environmental factors that may have an important regulating role.

*Trichostrongylus retortaeformis* represents the second-most prevalent parasite detected. The high prevalence might be favored, as for coccidia, by the direct cycle, with a fecal-oral transmission route. This nematode is commonly detected in hares [30,31] and can induce chronic enteritis within the duodenum and jejunum [6] with a reduction in health, body condition and reproductive potential [32].

In Italy the prevalence of *T. retortaeformis* infections varied a lot, ranging from very low (3%) [8] to extremely high (75%) [33]; however, the rates of positivity detected in other European countries showed prevalence constantly over 50% [6,7,21], both in captive and free-ranging animals. An overview of the most relevant national and European data referring to *T. retortaeformis* detected in European brown hare populations is provided in Table 4.

*Passalurus ambiguous* has also been isolated with a significantly lower prevalence (9.3%) than *T. retortaeformis*. This oxyurid is a parasite of lagomorphs, inhabiting the caecum and colon without triggering clinical manifestations; it is rarely detected in hares, but much more common in wild rabbits (*Oryctolagus cuniculus* Linnaeus, 1758).

The only tapeworm isolated was *Cittotenia* spp., whereas other national studies highlighted also the presence of the genus *Andrya* [11]. In the analyzed captive population, lungworms of the genus *Protostrongylus* were not found, despite being reported in hares from a wild, but managed, population within a regional park in Central Italy [11], as well as in several other reports across Europe with medium-to-high prevalence, especially in Austria (26.3%) and in Czech Republic (30.9%) [6]. An explanation of the absence of lungworms from the captive-bred population of our survey might be due to the habitat recreated inside the enclosure that is not particularly favorable for terrestrial gastropods, which act as intermediate hosts.

Analogous conclusions may be drawn to justify the scarce prevalence, 1.4% (3/215), of the trematode *D. dendriticum*, whose eggs have been detected in only one (A1) out of the eight breeding facilities. Besides, along with the gastropod host, *D. dendriticum* would also require the presence of infected ants to fulfill its biological cycle in the definitive host. According to Chorust et al. [6], *D. dendriticum* infections can be found in hares living in sympatry with large ruminants such as sheep and cattle. Poli et al. [31] first reported the parasite in Italian hares with a prevalence of 1.3% (1/72); whereas, a higher prevalence has been reported in other European countries such as Macedonia and Greece (9.5%) [7] or Spain (11%) [21].

## 5. Conclusions

The present study investigated a significant population of hares bred in Southern Italy and contributes to increase knowledge of the health status of a scantly investigated species, being also useful to optimize breeding effort in captivity.

Similar surveys would also be beneficial in the management and conservation of both the congeneric Italian hare (*Lepus corsicanus* de Winton, 1898), an endemic species distributed in scattered nuclei in Central and Southern Italy in Sicily and in Corsica, and of the Sardinian hare (*Lepus capensis mediterraneus* Wagener, 1841), an insular endemism of Sardinia. Overlooking parasitological analyses can be extremely detrimental for captive breeding efforts that aim to create a viable population for the conservation of vulnerable and declining species such as the Italian hare species.

## Figures and Tables

**Table 1:** Overall and relative prevalence of parasitic species in each hare-breeding facility.

Parasite	N. Positive Pools/N. Analyzed Pools (%, 95% C.I.)								Overall Prevalence (%, 95% C.I.)
	A1	A2	A3	A4	A5	A6	A7	A8	
* *Cittotenia* spp.	6/27 (22.2%, 8.6–42.3)	0/21 (0%, 0–16.1)	0/32 (0%, 0–10.9)	0/39 (0%, 0–9)	3/30 (10%, 2.1–26.5)	3/21 (14.3%, 3–36.3)	0/40 (0%, 0–8.8)	0/5 (0%, 0–52.2)	12/215 (5.6%, 2.9–9.5)
* *Coccidia*	24/27 (87.5%, 70.8–97.6)	21/21 (100%, 83.9–100)	26/32 (81.3%, 63.6–92.8)	39/39 (100%, 91–100)	30/30 (100%, 88.4–100)	19/21 (90.5%, 69.6–98.8)	33/40 (82.5%, 67.2–92.7)	5/5 (100%, 47.8–100)	197/215 (91.2%, 87.1–95)
* *Dicrocoelium* *dendriticum*	3/27 (11.1%, 2.4–29.2)	0/21 (0%, 0–16.1)	0/32 (0%, 0–10.9)	0/39 (0%, 0–9)	0/30 (0%, 0–11.6)	0/21 (0%, 0–16.1)	0/40 (0%, 0–8.8)	0/5 (0%, 0–52.2)	3/215 (1.4%, 0.2–5)
** *Passalurus* *ambiguus*	0/27 (0%, 0–12.8)	5/21 (23.8%, 8.2–47.2)	0/32 (0%, 0–10.9)	2/39 (5.1%, 0.6–17.3)	3/30 (10%, 2.1–26.5)	5/21 (23.8%, 8.2–47.2)	5/40 (12.5%, 4.2–26.8)	0/5 (0%, 0–52.2)	20/215 (9.3%, 5.8–14)
* *Strongyloides* *papillosus*	5/27 (18.5%, 6.3–38.1)	3/21 (14.3%, 3–36.3)	4/32 (12.5%, 3.5–29)	0/39 (0 %, 0–9)	0/30 (0%, 0–11.6)	0/21 (0%, 0–16.1)	2/40 (5%, 0.6–16.9)	0/5 (0%, 0–52.2)	14/215 (6.5%, 3.6–10.7)
** *Trichostrongylus retortaeformis*	7/27 (26%, 11.1–46.3)	7/21 (33.3%, 14.6–57)	10/32 (31.3%, 16.1–50)	4/39 (10.3%, 2.9–24.2)	5/30 (16.6%, 5.6–34.7)	6/21 (28.6%, 11.3–52.2)	5/40 (12.5%, 4.2–26.8)	2/5 (40%, 5.3–85.3)	46/215 (21.4%, 16.1–27.5)

95% Confidence Interval (C.I.). * No statistically significant difference among breeding facilities (*p* > 0.05). ** Statistically significant difference among breeding facilities (*p* < 0.05).

**Table 2:** Overall and relative prevalence of coccidia from the Genus *Eimeria* in each hare-breeding facility.

Coccidian Species	N. Positive Pools/N. Analyzed Pools (%, 95% C.I.)								Overall Prevalence (%, 95% C.I.)
	A1	A2	A3	A4	A5	A6	A7	A8	
*E. europaea*	0/27 (0%, 0–12.8)	5/21 (23.8%, 8.2–47.2)	0/32 (0%, 0–10.9)	13/39 (33.3%, 19.1–50.2)	5/30 (16.6%, 5.6–34.7)	0/21 (0%, 0–16.1)	0/40 (0%, 0–8.8)	0/5 (0%, 0–52.2)	23/215 (10.7%, 6.9–15.6)
*E. hungarica*	0/27 (0%, 0–12.8)	3/21 (14.3%, 3–36.3)	5/32 (15.6%, 5.3–32.8)	0/39 (0%, 0–9)	0/30 (0%, 0–11.6)	6/21 (28.6%, 11.3–52.2)	0/40 (0%, 0–8.8)	0/5 (0%, 0–52.2)	14/215 (6.5%, 3.6–10.7)
*E. l. leporis*	20/27 (74%, 53.7–88.9)	20/21 (95.2%, 76.2–99.9)	25/32 (78.1%, 60–90.7)	30/39 (77%, 60.7–88.9)	0/30 (0%, 0–11.6)	19/21 (90.5%, 69.6–98.8)	30/40 (75%, 58.8–87.3)	5/5 (100%, 47.8–100)	149/215 (69.3%, 62.7–75.4)
*E. robertsoni*	20/27 (74%, 53.7–88.9)	21/21 (100%, 83.9–100)	17/32 (53.1%, 34.7–70.9)	30/39 (77%, 60.7–88.9)	16/30 (53.3%, 34.3–71.7)	8/21 (38.1%, 18.1–61.6)	20/40 (50%, 33.8–66.2)	2/5 (40%, 5.3–85.3)	134/215 (62.3%, 55.5–68.8)
*E. semisculpta*	10/27 (37.5%, 19.4–57.6)	0/21 (0%, 0–16.1)	5/32 (15.6%, 5.3–32.8)	4/39 (10%, 2.9–24.2)	8/30 (26.7%, 12.3–45.9)	8/21 (38.1%, 18.1–61.6)	5/40 (12.5%, 4.2–26.8)	0/5 (0%, 0–52.2)	4/215 (18.6%, 13.6–24.5)
*E. townsendi*	17/27 (62.5%, 42.4–80.6)	17/21 (80.9%, 58.1–94.6)	17/32 (53.1%, 34.7–70.9)	24/39 (61.5 %, 44.6–76.6)	26/30 (86.7%, 69.3–96.2)	13/21 (61.9%, 38.4–81.9)	13/40 (32.5%, 18.6–49.1)	2/5 (40%, 5.3–85.3)	129/215 (60%, 53.1–66.6)

95% Confidence Interval (C.I.).

**Table 3:** Prevalence of coccidia from the Genus *Eimeria* in European hares across Europe.

Infection Prevalence, Data Source *							
Ita1 ^a^	Ita2 ^b^	Ita3 ^c^	Aus ^d^	Cze ^e^	Gre/Mac ^f^	Spa ^g^	Bul ^h^
91.2%, CB	34.8%, CB 87.7%, W	34.8%, CB 87.7%, W	80.4%, W	79.6%, W	64.3%, W	71.7%, W	55.3%, W

*: CB = captive bred, W = wild; a: this survey; b: [11]; c: [8]; d, e: [6]; f: [7]; g: [21]; h: [10].

**Table 4:** Prevalence of *Trichostrongylus retortaeformis* in European hares across Europe.

Infection Prevalence, Data Source *							
Ita1 ^a^	Ita2 ^b^	Ita3 ^c^	Ita4 ^d^	Aus ^e^	Cze ^f^	Gre/Mac ^g^	Spa ^h^
21.4%, CB	72%, W	65%	3%, CB 87.1%, W	82.7%, W	83.2%, W	50%, W	56.6%, W

*: CB = captive bred, W = wild; a: this survey; b: [31]; c: [30]; d: [8]; e, f: [6]; g: [7]; h: [21].

## Data Availability

Data is available from the corresponding Author upon reasonable request.
